# A Transformer-Based Bridge Structural Response Prediction Framework

**DOI:** 10.3390/s22083100

**Published:** 2022-04-18

**Authors:** Ziqi Li, Dongsheng Li, Tianshu Sun

**Affiliations:** School of Civil Engineering, Dalian University of Technology, Dalian 116024, China; 496092015@mail.dlut.edu.cn (Z.L.); tssun@dlut.edu.cn (T.S.)

**Keywords:** bridge structural response prediction, transformer, deep learning, structural health monitoring, encoder–decoder

## Abstract

Structural response prediction with desirable accuracy is considerably essential for the health monitoring of bridges. However, it appears to be difficult in accurately extracting structural response features on account of complex on-site environment and noise disturbance, resulting in poor prediction accuracy of the response values. To address this issue, a Transformer-based bridge structural response prediction framework was proposed in this paper. The framework contains multi-layer encoder modules and attention modules that can precisely capture the history-dependent features in time-series data. The effectiveness of the proposed method was validated with the use of six-month strain response data of a concrete bridge, and the results are also compared with those of the most commonly used Long Short-Term Memory (LSTM)-based structural response prediction framework. The analysis indicated that the proposed method was effective in predicting structural response, with the prediction error less than 50% of the LSTM-based framework. The proposed method can be applied in damage diagnosis and disaster warning of bridges.

## 1. Introduction

Response data of bridge structures can be used to participate in the assessment of structural health, and when the response values are above a certain range of the norm, it indicates that the monitored bridge structure is at risk of abnormalities or damage [1,2,3,4]. In this context, there is a need to be able to accurately predict the response of bridge structures. Structural response data are a kind of time-series data, which are usually used to predict the future period of data from a certain period of time in the past. These kinds of data have been widely used in economic and social science fields, such as weather prediction, stock prediction, etc. Traditional structural response prediction methods mainly focus on some linear and nonlinear models [5,6,7,8], which have good results in some simple systems, but for high-order nonlinear systems with long-term time dependence and spatial correlation, these methods have the drawbacks of huge computational effort and insufficient accuracy [9].

Early neural networks mainly refer to Back Propagation (BP) neural networks [10], and these networks were used in areas such as predicting financial markets [11,12], electrical loads [13,14,15], and traffic accidents [16,17]. These networks have a common problem: the output data are only related to the input data, but not the order of the input data. The neurons themselves do not have the ability to store information, and the whole network has no “memory” capability. With the rise of deep learning, deep learning networks have shown good performance in speech recognition and image processing [18,19,20], and LSTM is considered to be the best network model for processing time-series data [21,22]. Therefore, LSTM has gradually become a research hotspot in the field of engineering structures, especially in the operation and maintenance phase of bridges, where it can predict the response of bridges in a short period of time, and many researchers have proposed LSTM-based response prediction models. Zhang et al. [23] established a convolutional long-short term memory (ConvLSTM) network to learn spatiotemporal latent features from data, and thus establish a surrogate model for structural response forecasting. Li et al. [24] applied LSTM to model the bridge aerodynamic system with the potential fluid memory effect. Bilal et al. [25] developed a LSTM network with overlapping data to evaluate important response data after earthquakes. However, LSTM still has its limitations, and the semantic capture capability for time-series data is still insufficient, which may lead to poor prediction accuracy. To remedy this deficiency, researchers have proposed the Transformer structure [26], which is better than LSTM in the semantic capture of time-series data due to the use of an attention mechanism as the underlying network.

Therefore, in this paper, a Transformer-based bridge structural response prediction framework is proposed for improving the accuracy of bridge structural response prediction, and the performance of the proposed framework is tested on a concrete bridge. To the best of our knowledge, this is the first Transformer framework that has been used for bridge structural response prediction. The paper is organized as follows. Section 2 provides the details of the proposed framework. Section 3 provides basic information about the bridge response prediction experiments, containing the dataset, training parameters, etc. Section 4 provides the experimental results. Section 5 provides the discussion. Section 6 provides the limitations of the proposed method. Section 7 provides the conclusion.

## 2. Framework for Structural Response Prediction

The traditional CNN and RNN are discarded in Transformer, and the whole network structure is composed entirely of an attention mechanism. The original Transformer consists of and only consists of Self-Attention and Feed Forward Neural Network. After years of development, Transformer has produced many variants.

**Figure 1:**
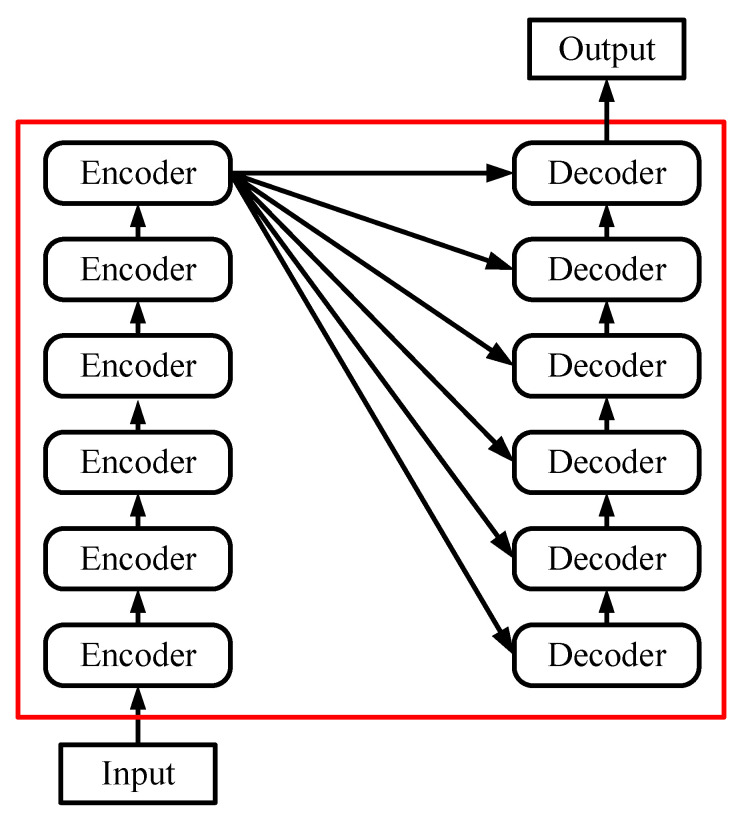
Structure of the used Transformer.

The Transformer structure used in this paper is shown in the Figure 1, with an encoder–decoder structure. The encoder consists of 6 encoding blocks, and similarly the decoder is composed of 6 decoding blocks. As with all generative models, the output of the encoder will be used as the input to the decoder.

### 2.1. Attention Mechanism

Attention mechanisms [27] have been widely used in various areas of deep learning in recent years, and is easily encountered in various different types of tasks, be it image processing, speech recognition, or natural language processing. Its inspiration comes from the human attention mechanism. The visual attention mechanism is a signal processing mechanism in the brain that is unique to human vision. By quickly scanning the global image, human vision obtains the target area to focus on, which is generally known as the focus of attention, and then devotes more attention resources to this area to obtain more detailed information about the target to be focused on, while suppressing other useless information. [28,29]. Its purpose is to select the information that is more critical to the current task goal from among the many information available.

The input vector is denoted by $X=[X_{1},X_{2},\ldots ,X_{n}]$. In order to match a weight to the input vector, the attention weight $\mathrm{Attention}=[a_{1},a_{2},\ldots ,a_{n}]$ is calculated as follows:

(1) $$\mathrm{Attention}=\mathrm{Softmax}\;(\frac{QK^{T}}{\sqrt{d_{k}}})V$$

where *Q*, *K*, and *V* denote “query”, “key”, and “value”, respectively; *d_k_* is the scaling factor and denotes the dimensionality of *K*. For larger values of *d_k_*, the product of dot products is too large, thus pushing the Softmax function to regions with very small gradients. To counteract this effect, the dot product is scaled using $\frac{1}{\sqrt{d_{k}}}$.

After getting Attention, it will be sent to the next module of encoder, i.e., feed forward neural network. This is fully connected and has two layers: the first layer has an activation function of ReLU, and the second layer is a linear activation function that can be expressed as:

(2) $$\mathrm{FFN}(\mathrm{Attention})=\max(0,\;\mathrm{Attention}W_{1}+b_{1})W_{2}+b_{2}$$

**Figure 2:**
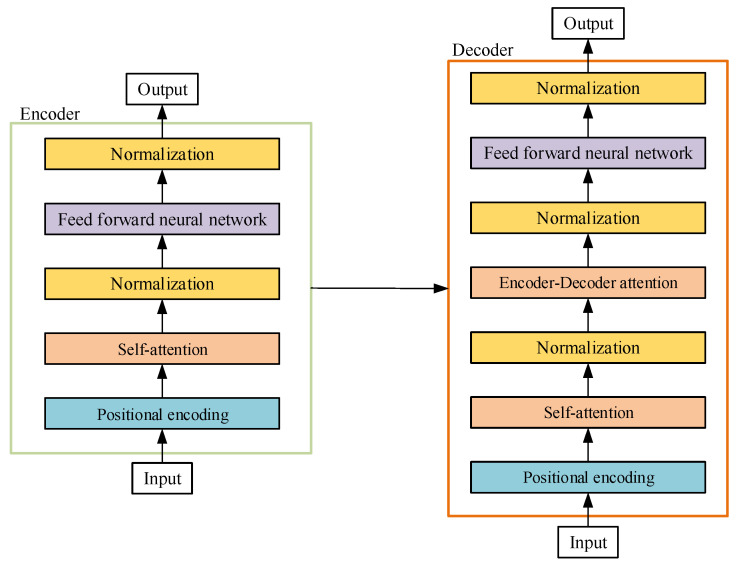
Internal Structure of Encoder and Decoder.

As shown in Figure 2, there are two kinds of attention mechanisms used in Transformer, Self-Attention and Encoder–Decoder attention. Both are computed in a multi-head way, but Encoder–Decoder attention uses the traditional attention mechanism, where Query is the encoder value at the last time i had been computed by Self-Attention, and both Key and Value are the output of Encoder. Self-Attention only calculates the attention (or weight matrix) inside the encoder or decoder without reference to the current state at the decoder’s side.

### 2.2. Positional Encoding

Since there is no loop nor convolutional structure in the Transformer, in order for the model to be able to utilize the order of the sequence, it is necessary to insert some information that can represent the relative or absolute position in the sequence [30]. Position encoding is not part of the model architecture, it is actually only part of the preprocessing. For each position vector, it provides a unique encoding. The dimensionality of the encoding used in this paper is 250 dimensions.

### 2.3. Multi-Head Attention

Multi-Head attention [31] provides multiple “representation subspaces” for an attention. Because different Query/Key/Value weight matrices are used in each attention, each matrix is generated by random initialization. Then, through training, the response values are projected into different “representation subspaces”. Multi-Head attention in this paper consists of self-attention stacking to form a depth structure. The calculation is shown as follows:

(3) $$Q_{i}=QW_{i}^{Q},K_{i}=KW_{i}^{K},V_{i}=VW_{i}^{V},i=1,\ldots ,8$$

(4) $${\mathrm{head}}_{i}=\mathrm{Attention}(Q_{i},K_{i},V_{i}),i=1,\ldots ,8$$

(5) $$\mathrm{MultiHead}(Q,K,V)=\mathrm{Contact}({\mathrm{head}}_{1},\ldots ,{\mathrm{head}}_{8})W^{O}$$

In this paper, the $Q,K,V\in R^{250},W_{i}^{Q},W_{i}^{K},W_{i}^{V}\in R^{250\times 64},W^{O}\in R^{250\times 250},{\mathrm{head}}_{i}\in R^{64}$.

## 3. Experiment

In this paper, our objective is to predict the bridge response for a specific period in the future based on the bridge response for a given period in the past, where the main load on the bridge is the vehicle load, and the bridge response used in this paper is the strain. We established a dataset of bridge strains, and the specific information of this dataset is shown in the description later. To show the performance of our proposed method, we compare the proposed method with previous LSTM-based methods.

### 3.1. Songhua River Bridge Structural Response Dataset

**Figure 3:**
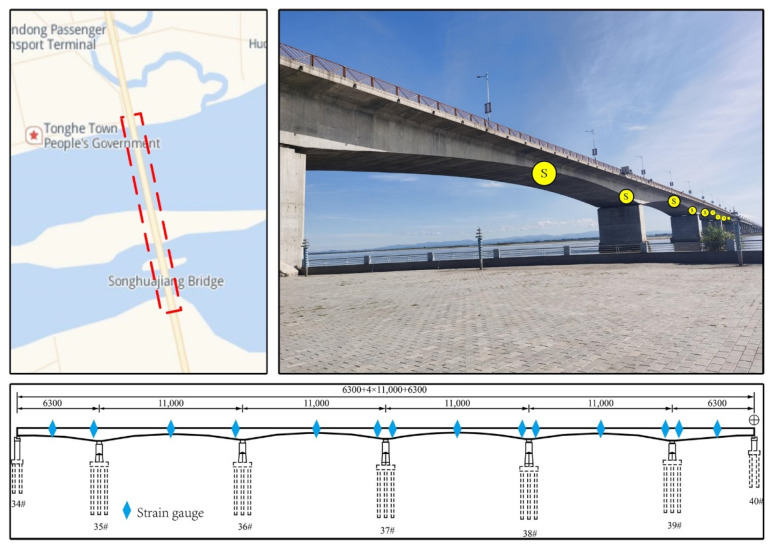
Location of Songhua River Bridge and sensor layout.

This bridge is located in Tonghe County, Heilongjiang Province. The total length of the bridge is 2578.28 m. The main bridge structure is a prestressed concrete continuous box girder divided into two links. Each link span arrangement is 1132 m, and 14 sensors were placed on each link span (From left to right, S01–S14). The strain responses of four sensors (S01, S02, S03, and S09) were selected as the dataset in this paper. The reason for our arrangement of the 14 sensors was due to the need of other projects, and this number of sensors was not needed for the study in this paper. The method in this paper has good prediction for each sensor, only four sensors were randomly selected. The strain response was monitored for 6 months, and was measured every half hour. The sensor layout is shown in Figure 3.

### 3.2. Training Platform

The training process was performed on a single workstation using a high-performance GPU (NVIDIA RTX 2080Ti) and CPU (AMD Ryzen 2700X 3.7 GHz). The code was written in Python 3.6, the framework was built using Pytorch 1.8.0, and the training process was performed on Windows 10. The optimizer was Adam with a learning rate of 0.001 and a decay rate of 0.0001. Epoch was set to 100.

### 3.3. Loss Function and Evaluation Metrics

RMSE (Root Mean Square Error): RMSE is the most commonly used regression loss function as well as evaluation metrics; it is calculated by finding the square root of the sum of squares of the distance between the predicted and true values, with the following formula:

(6) $$RMSE=\sqrt{\frac{1}{N}\sum_{i=1}^{n}{\left(y_{i}-{\hat{y}}_{i}\right)}^{2}}$$

where $y_{i}$ is the true value, ${\hat{y}}_{i}$ is the predicted value, and *n* is the number of samples.

### 3.4. Experimental Design

In this paper, two deep learning methods were used for strain response prediction; the first one is the proposed Transformer-based method and the second one is the LSTM-based method. The dataset was used as the bridge strain response dataset in Section 3.1. Both methods used the same training parameters, with the strain response of the previous two hours as the input and the strain response of the next half hour as the prediction target, and the epochs were set to 100. The ratio of training set, validation set, and test set was 0.7:0.1:0.2.

## 4. Experimental Results

**Figure 4:**
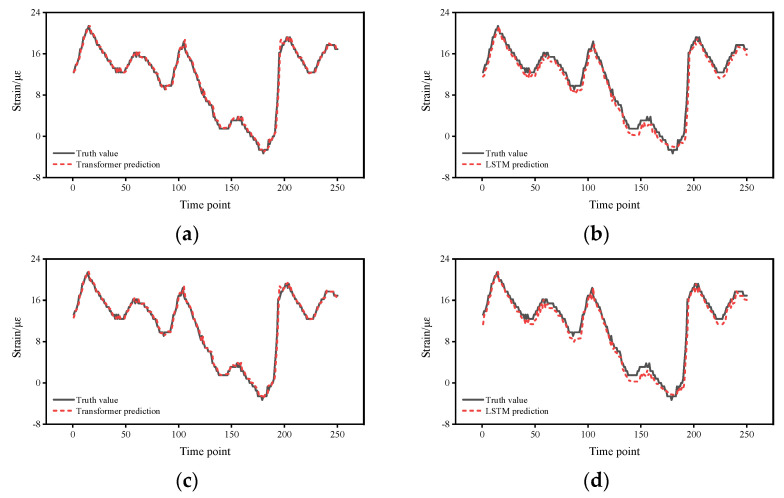

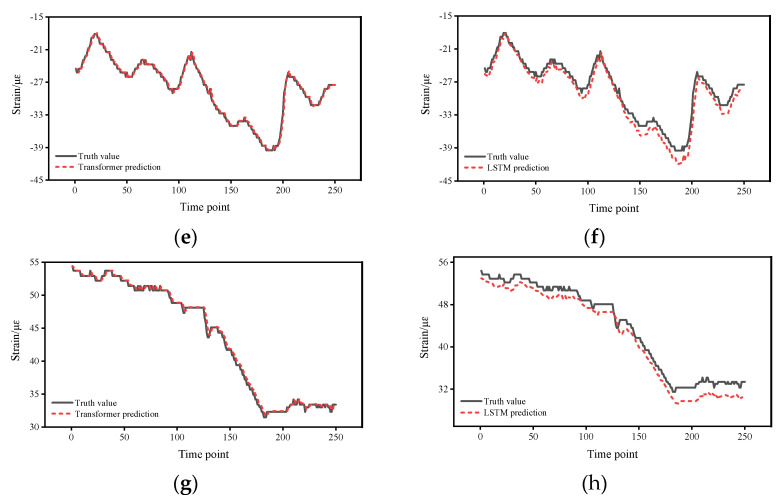
Strain response prediction results of the two methods: (**a**) Transformer prediction for S01. (**b**) LSTM prediction for S01. (**c**) Transformer prediction for S02. (**d**) LSTM prediction for S02. (**e**) Transformer prediction for S03. (**f**) LSTM prediction for S03. (**g**) Transformer prediction for S09. (**h**) LSTM prediction for S09.

Figure 4 shows the strain response prediction results of the two methods. From the prediction results of the Transformer, the strain response was successfully predicted and the predicted strain response values basically matched with the field test values, indicating that the proposed method is able to predict the strain response in the short term. From the comparison results, the agreement of the proposed method is better than that of the LSTM, which, in general, predicts the strain response trend of the structure, but from the details, there is still a significant difference between the predicted strain response and the test value at some time points, indicating that the stability of the LSTM prediction is not as good as that of the proposed method. To better show the details of the errors, the variation of the errors with time points and the probability density function are calculated so that the variation of the errors with time points and the distribution of the errors can be more accurately reflected.

**Figure 5:**
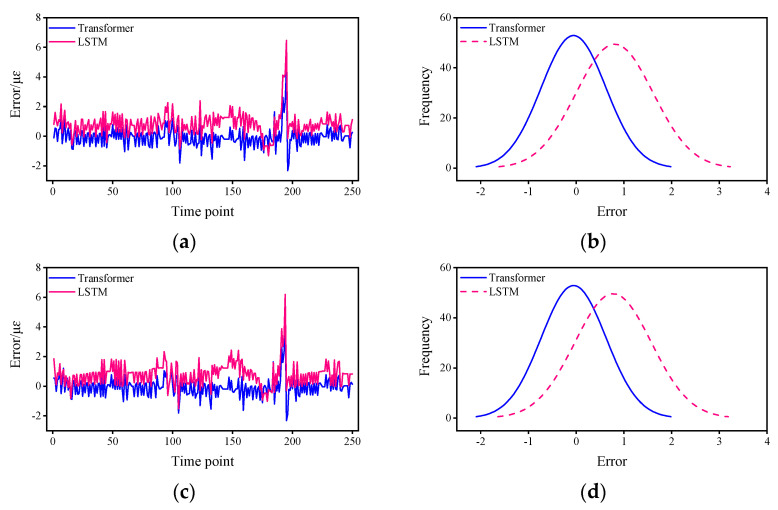

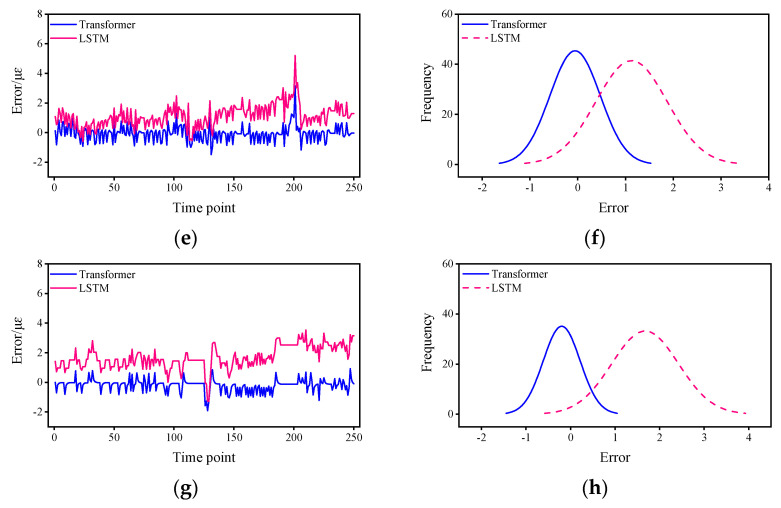
Prediction error statistics and error distribution for Transformer and LSTM: (**a**) Error curve of sensor S01. (**b**) Fitting curve of normal distribution of sensor S01. (**c**) Error curve of sensor S02. (**d**) Fitting curve of normal distribution of sensor S02. (**e**) Error curve of sensor S03. (**f**) Fitting curve of normal distribution of sensor S03. (**g**) Error curve of sensor S09. (**h**) Fitting curve of normal distribution of sensor S13.

The Figure 5 shows the errors of the Transformer-based method and LSTM, as well as the fitted curves of the normal distribution of their error fits. From the error curves, it can be seen that the error of Transformer is smaller than that of LSTM, and both have the same trend of change, with jitter occurring at the 200th time point. Table 1 shows the mean error and the 95% confidence interval (CI). From the mean value of the error, the mean error of the Transformer is about 19.2–55.5% of that of the LSTM. From the 95% CI, there is a much narrower CI for the Transformer of approximately 59.0–87.7% for the LSTM. The strain responses used in this study are all raw data without pre-processing such as filtering, and the error is controlled within an acceptable range in the presence of noise, which shows the engineering feasibility of the proposed method.

## 5. Discussion

### 5.1. Impact of Different Number of Prediction Points

**Figure 6:**
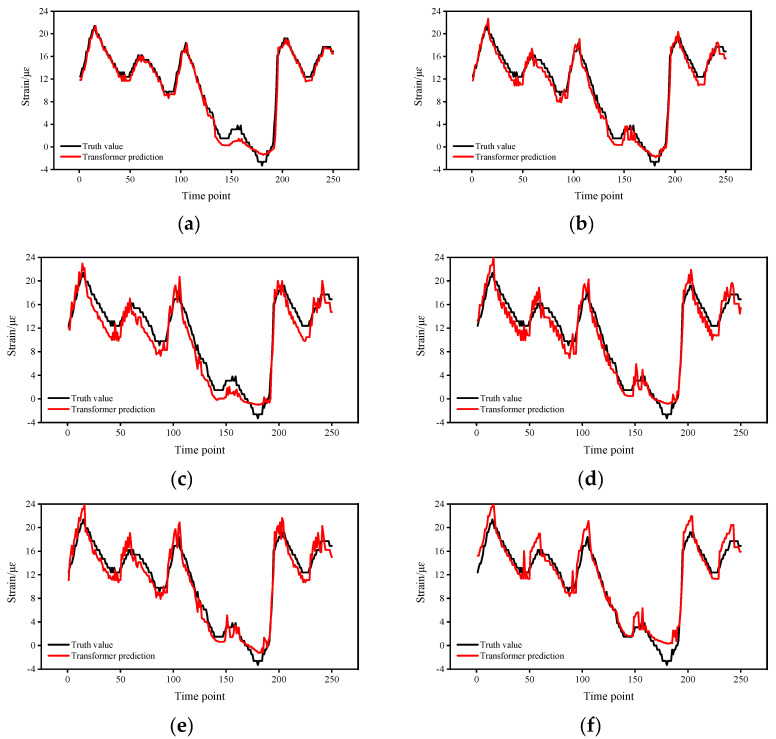
Prediction results for different numbers of prediction points: (**a**) Two-point prediction. (**b**) Four-point prediction. (**c**) Six-point prediction. (**d**) Eight-point prediction. (**e**) Ten-point prediction. (**f**) Twelve-point prediction.

To test the performance of the proposed method in predicting different numbers of strain responses, we changed the parameters of the sliding window and adjusted the number of prediction points to two, four, six, eight, ten, and twelve, respectively, and the results are shown in Figure 6. In particular, when the number of prediction points is greater than one, multiple batches of prediction values are generated for each time point, and the mean value of multiple prediction values is used as the final prediction value in this paper. The RSME for each of the six cases are calculated, and it indicates the magnitude of the prediction error, and the larger the RSME, the larger the prediction error.

**Figure 7:**
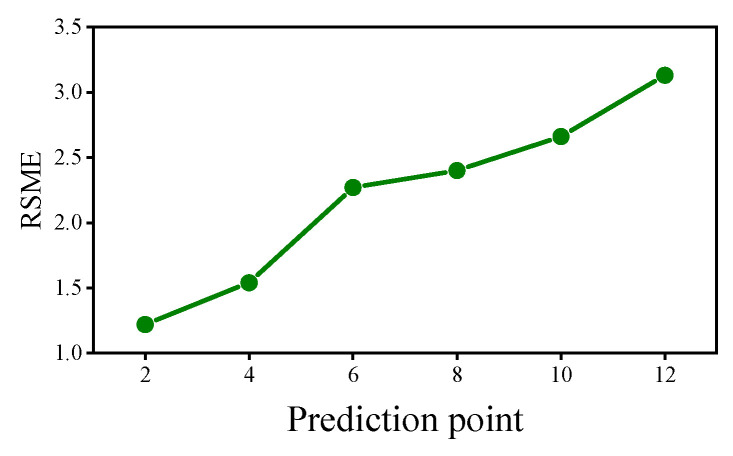
Prediction error for different number of prediction points.

The prediction errors are counted in Figure 7. The results show that the proposed methods can predict the strain response when the prediction value is less than twelve points, but the accuracy varies widely. As the number of prediction points increases, the prediction error becomes larger. When the number of prediction points is four, the RSME increases significantly; when the number of prediction points increases to six, the increase in RSME becomes flat; when the number of prediction points is ten, the increasing trend of RSME becomes faster. Combining the results of Figure 6 and Figure 7, when the number of prediction points is within four points, the prediction error is small, and the prediction results are more credible; when the number of prediction points is between four and ten, the prediction error is moderate, and the results are less credible; when the number of prediction points is greater than ten, the prediction error is relatively large, and the results are not credible.

### 5.2. Impact of Different Time Intervals

In this section, the effect of different time intervals on the prediction results is discussed, and the dataset is set to a uniform length of 2000 samples in order to demonstrate fairness. After our experiments, it was found that the length of the batch had a large impact on the training results. In previous tests, the batch was set to 32, and the previous conclusions are based on this setting. In this section, when the batch was set to 32, the prediction accuracy was found to drop sharply. As a result, the length of the batch was reduced to six for multiple tests, and the prediction accuracy dropped more slowly when the time interval was increased at this point.

**Figure 8:**
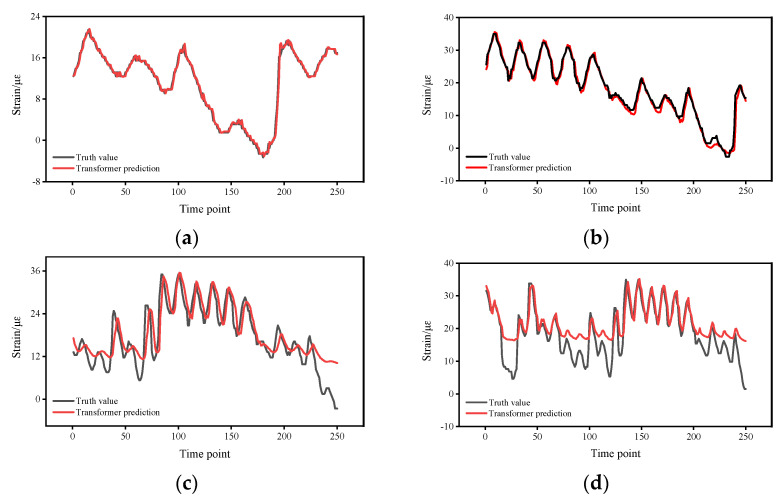
Prediction results at different time intervals. (**a**) 0.5 h. (**b**) 1 h. (**c**) 1.5 h. (**d**) 2 h.

**Figure 9:**
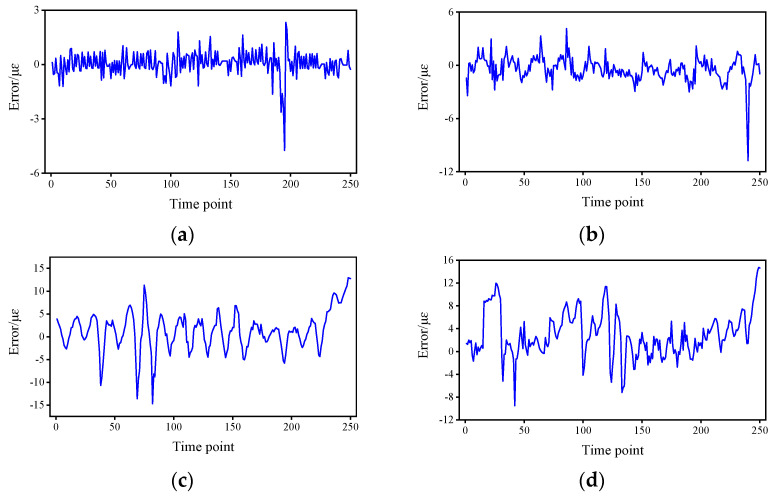
Prediction errors at different time intervals. (**a**) 0.5 h. (**b**) 1 h. (**c**) 1.5 h. (**d**) 2 h.

Figure 8 shows the prediction results and errors of the Transformer at different time intervals, which were set to 0.5 h, 1 h, 1.5 h, and 2 h. The results show that, in general, the prediction errors gradually increase as the time interval increases. Figure 9 shows the prediction errors of different time intervals; it can be seen the prediction results are more reliable for time intervals of 0.5 h and 1 h, and the prediction errors are mainly distributed in (−3, 3) and (−6, 3), while the prediction results are less reliable for time intervals of 1.5 h and 2 h, and the main distribution intervals of the errors are in (−15, 15) and (−12, 16). According to the prediction results, it can be seen that the time interval has a large influence on the prediction accuracy, and the prediction accuracy decreases significantly when the time interval is increased from 1 to 1.5 h, indicating that the response data of the bridges are more regular when the time interval is small, which is related to the form of external loads on the bridges and the local traffic conditions. Therefore, in practical applications, it is best to keep the time interval at a low level, as much as possible within 1 h.

## 6. Limitations

**Figure 10:**
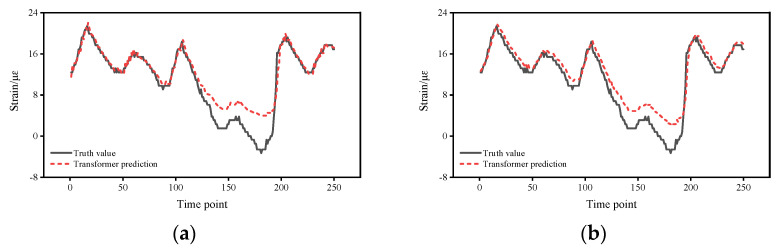

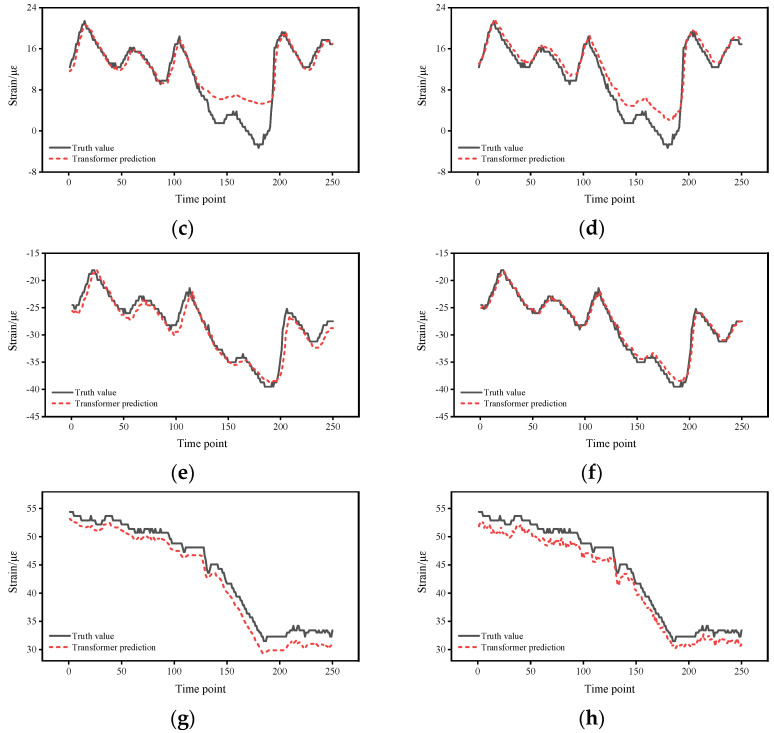
The prediction results of Transformer and LSTM when the input points are 3 points: (**a**) Transformer prediction for S01. (**b**) LSTM prediction for S01. (**c**) Transformer prediction for S02. (**d**) LSTM prediction for S02. (**e**) Transformer prediction for S03. (**f**) LSTM prediction for S03. (**g**) Transformer prediction for S09. (**h**) LSTM prediction for S09.

This section analyzes the limitations of the proposed method. Since there are various options for the number of input points and number of output points for structural response prediction, the use case presented in the previous section is the best application case with both four points for input and one point for prediction. However, as the input points change, not all cases are more accurate for Transformer than LSTM, so this section changes the input points to three points to compare the effects of the two prediction methods. The results are shown in Figure 10. In the case of three input points, the prediction accuracy of Transformer for S01, S02, and S09 is slightly lower than that of LSTM, and the prediction accuracy of Transformer for S03 is almost the same as that of LSTM. In contrast to the case with four input points, the prediction accuracy of Transformer for all sensors with three input points is less than the former, while the prediction accuracy of LSTM for S01, S02, and S09 decreases, and increases for S03. After several tests, the prediction result tends to be stable after increasing the input points, which is similar to the prediction result in Figure 4, so it is recommended to use at least four points as input points in order to ensure stable prediction results.

## 7. Conclusions

In this paper, a Transformer-based time series prediction framework is proposed for predicting the structural response of bridges with time dependence. The proposed framework contains multiple encoder modules and attention modules, and this structure enhances the semantic recognition of the temporal series data and is more conducive to extracting the features of the structural response. The accuracy of the proposed framework is verified by six-month strain response data of a concrete bridge. The proposed framework is compared with the most commonly used LSTM-based structural response prediction framework, and the results is shown as follows:

- From the mean value of the error, the mean error of the Transformer is about 19.2%–55.5% of that of the LSTM.
- From the 95% CI, with a much narrower CI for the Transformer of approximately 59.0%–87.7% for the LSTM.

Deep learning-based structural response prediction has the drawback of poor interpretability. Compared with traditional methods, deep learning-based methods rely more on the temporal regularity features of the data itself, which mainly reflects the approximation ability of deep learning and cannot correspond to the real physical features. In the future, we will pay more attention to the interpretability of time-series prediction, and calculate which time points of data are more valuable for predicting future responses.

## Figures and Tables

**Table 1:** Mean error and 95%CI statistical results.

Sensor No.	Mean Error		95%CI	
	Transformer	LSTM	Transformer	LSTM
1	0.48	0.90	(−1.29, 1.28)	(−0.79, 2.14)
2	0.48	0.85	(−1.29, 1.28)	(−0.75, 2.26)
3	0.37	1.12	(−1.03, 0.89)	(−0.35, 2.64)
9	0.31	1.61	(−1.04, 0.70)	(0.35, 3.30)
